# MEDIA REPRESENTATION OF OLDER WOMEN DURING WAR CONFLICT: CASE OF UKRAINE AND ISRAEL

**DOI:** 10.1093/geroni/igae098.4331

**Published:** 2024-12-31

**Authors:** Ksenya Shulyaev, Tova Band-Winterstein

**Affiliations:** University of Haifa, Haifa, HaZafon, Israel; University of Haifa, Haifa, HaZafon, Israel

## Abstract

Media reportage of war conflicts tends to underrepresent women, especially older women, and distort their involvement in wars through narrow role characterizations. Women are often cast less as political actors and more as helpless victims. This backdrop sets the stage for our study, which examines the media portrayal of older women during wartime, focusing on conflicts in Ukraine and Israel. By content-analyzing news from Ukraine and Israel, we identify recurring themes and highlight the roles and experiences of older women as depicted in media from the onset of the full-scale war in Ukraine (February 2022) and the “Iron Swords” in Israel (October 2022). The findings revealed five key portrayals of older women (1) Older Women as Victims: symbolizing the enemy’s inhumanity and brutality (2) Older Women as Enablers of Family Continuity: guardians of family bonds with warriors, maintaining household rituals and caring for grandchildren amidst chaos (3) Older Women as Resilient Heroines: Symbols of Hope, Survival and Legacy. (4) Older Women as Bearers of Wisdom: transmitting wisdom and stability during times of conflict. (5) Older Women as Symbols of Patriotism: Volunteers, donators, symbolizing endurance and national pride. The media’s portrayal of older women during wartime underscores their multifaceted roles and the profound impact of conflict on their lives and conveys symbolic messages to society to serve broader societal and political purposes. This study aims to contribute to reframing aging representation in media by enhancing knowledge, improving ageistic attitudes toward older women, and supporting addressing aging issues during wartime.

